# Wall Enhancement, Hemodynamics, and Morphology in Unruptured Intracranial Aneurysms with High Rupture Risk

**DOI:** 10.1007/s12975-020-00782-4

**Published:** 2020-01-20

**Authors:** Nan Lv, Christof Karmonik, Shiyue Chen, Xinrui Wang, Yibin Fang, Qinghai Huang, Jianmin Liu

**Affiliations:** 1 Department of Neurosurgery, Changhai Hospital, Second Military Medical University, Changhai Road 168, Shanghai, 200433 China; 2 MRI Core, Houston Methodist Research Institute, Houston, TX USA; 3 Department of Radiology, Changhai Hospital, Second Military Medical University, Shanghai, China

**Keywords:** Intracranial aneurysms, Hemodynamics, Morphology, Vessel wall imaging

## Abstract

The purpose of this study is to investigate the relationship between morphology, hemodynamics, and aneurysm wall enhancement (AWE) on vessel wall MRI and their potential role in rupture of intracranial aneurysms. Fifty-seven patients (22 males and 35 females; mean age of 58.4) harboring 65 unruptured intracranial aneurysms were retrospectively recruited. Vessel wall MRI images were reviewed and differentiated as no (NAWE), partial (PAWE), and circumferential (CAWE) wall enhancement. Computational geometry and computational fluid dynamics were used to calculate morphological and hemodynamic parameters. The PHASES score was calculated for each case to estimate its rupture risk. Univariate and multivariate logistic regression analysis was performed to investigate the relationship between morphological-hemodynamic pattern and AWE as well as their association with rupture risk. AWE was present in 26 (40.0%) lesions, including 14 (21.5%) PAWE and 12 (18.5%) CAWE. Aneurysm size (odds ratio = 7.46, 95% confidence interval = 1.56–35.77, *p* = 0.012), size ratio (odds ratio = 12.90, 95% confidence interval = 2.28–72.97, *p* = 0.004), and normalized wall shear stress (odds ratio = 0.11, 95% confidence interval = 0.02–0.69, *p* = 0.018) were independently associated with the presence of AWE. With increasing PHASES score, size-related parameters and the frequency of irregular shape increased significantly, and a hemodynamic pattern of lower and oscillating wall shear stress was observed. Simultaneously, the proportion of NAWE aneurysms decreased, and PAWE and CAWE aneurysms increased significantly (*p* < 0.001). Unruptured intracranial aneurysms with a higher rupture risk presented with a significantly larger size, lower wall shear stress, and more intense AWE, which might support the interaction between morphology, hemodynamics, and inflammation and their potential role in aneurysm rupture prediction.

## Introduction

Unruptured intracranial aneurysms (UIAs) occur in 3 to 5% of the general population, and increasing numbers are detected due to the wider availability of non-invasive imaging techniques [1, 2]. Both of microsurgery and endovascular treatment carry a non-negligible risk of procedural morbidity, which highlights the importance of understanding its pathogenesis to improve rupture risk evaluation [3]. Grading models for discriminating aneurysms with a high rupture risk were designed based on various factors as reported in previous studies. The PHASES score, derived from prospective studies, is one of the most discussed of these grading models [4, 5]. As an empirical quantity, it is solely based on clinical data and does not add to the understanding of the underlying mechanisms responsible for aneurysm evolution and rupture [6].

It is generally accepted that the evolution of intracranial aneurysms is driven by inflammation and progressive wall degradation [7, 8]. This hypothesis is supported by histologic analysis of resected human aneurysm tissue [7]. Aberrant hemodynamic conditions are believed to initiate a cascade of events, including inflammatory cell infiltration and cytokine accumulation [8, 9]. The lack of proper animal models and the difficulty of collecting human tissue samples impede studying the connections between the hemodynamic environment and aneurysmal wall characteristics.

The recent development of vessel wall magnetic resonance imaging (VW-MRI) provides a viable in vivo approach to investigate characteristic features of the aneurysmal wall. Several studies have suggested that aneurysmal wall enhancement (AWE) detected by VW-MRI is closely associated with the evolution of aneurysms and might be a potential marker for inflammatory reactions [10, 11]. Investigating the link between AWE and hemodynamics therefore presents an alternative in vivo method to improve our knowledge of the mechanisms for aneurysm pathogenesis.

In this study, we investigated the correlation of wall characteristics by VW-MRI and hemodynamic-morphological pattern calculated by computational methods, aiming to gain more evidence on the interaction of wall inflammation, hemodynamics, and morphology. In addition, the PHASES score was calculated to analyze the role of AWE, hemodynamics, and morphology in respect to aneurysm rupture risk prediction.

## Methods

The Institutional Review Board of Changhai Hospital approved this retrospective study and the requirement for informed consent was waived. The patients’ information was anonymized and de-identified prior to analysis.

### Population and Aneurysms

From January 2016 to December 2017, 153 consecutive patients with 231 unruptured IAs were diagnosed at our institution. The inclusion criteria for this study were (a) saccular UIAs with (b) VW-MRI images and (c) three-dimensional rotational angiography (3DRA) data. The exclusion criteria for the study were (a) aneurysms with uncertain rupture status; (b) UIAs other than saccular that is fusiform, traumatic, dissecting, and infectious; and (c) inadequate quality of 3DRA data for computational fluid dynamics (CFD) analysis. Finally, 57 patients with 65 UIAs (4 patients with double UIAs and 2 patients with triple UIAs) were included in this study.

### MRI Imaging Protocol and Analysis

MR imaging was performed using a 3.0-T MRI scanner (SIGNA 3.0 T, GE Healthcare, Milwaukee, USA) with an 8-channel head coil. Three-dimensional time-of-flight (TOF) magnetic resonance angiography (MRA) was performed for positioning. Then, T1-weighted black-blood fast spin-echo sequence was performed before and after contrast agent administration. The scanning parameters were as follows: TR/TE = 581 ms/20 ms, field of view (FOV) = 100 × 100 mm^2^, matrix = 256 × 320, echo train length = 6, resolution = 0.4 mm × 0.4 mm in-plane, slice thickness = 1.5 mm, and total scan time = 5 min. Post-contrast T1WI was performed immediately after intravenous injection of Gd-DTPA at a dose of 0.1 mmol/kg.

**Fig. 1:**
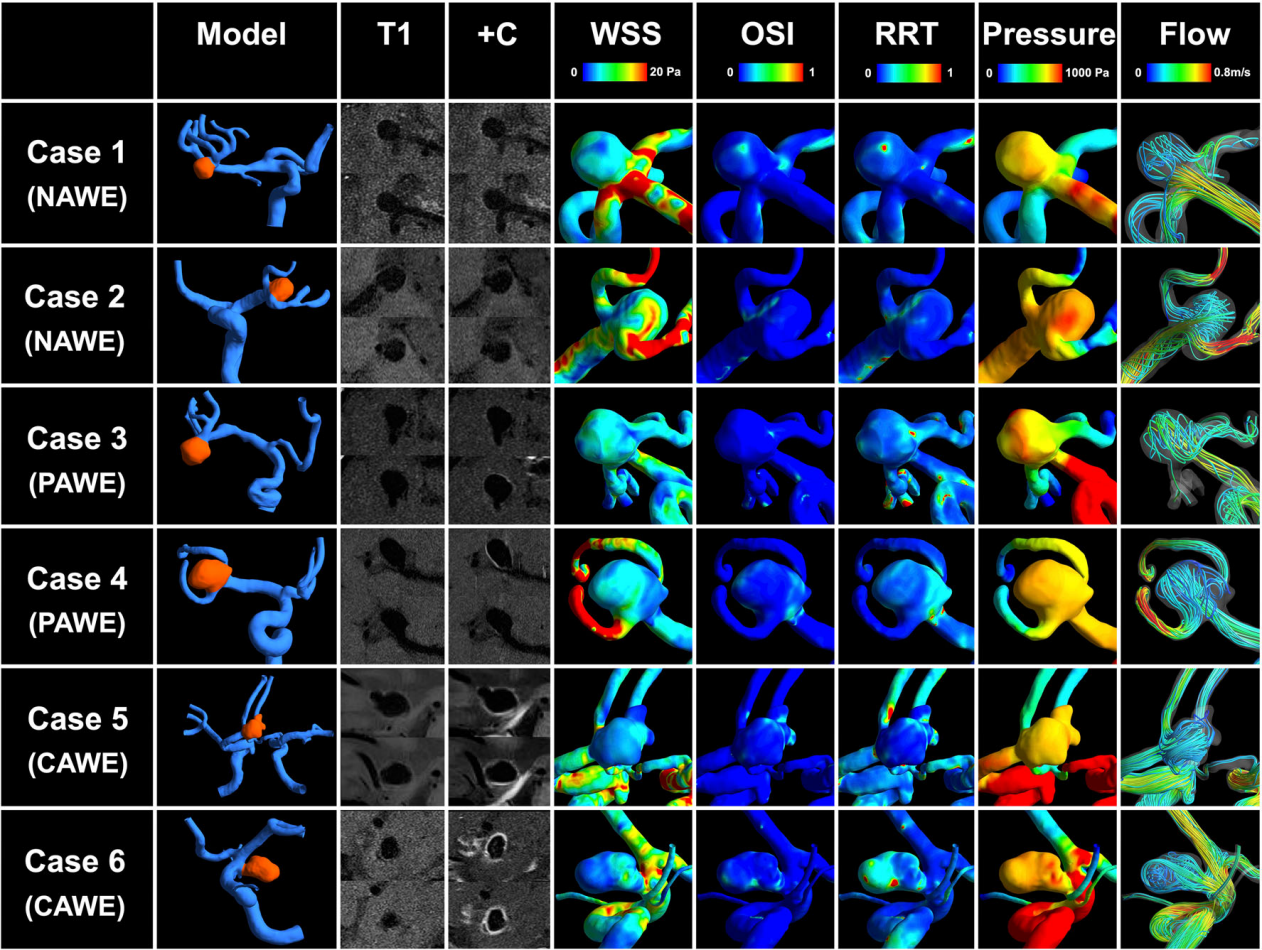
Presentative cases of aneurysmal wall enhancement and their hemodynamic patterns. Cases 1 and 2, no aneurysmal wall enhancement (NAWE) was observed by comparing the post-contrast images (+ C) to precontrast images (T1); cases 3 and 4, aneurysms with partial wall enhancement (PAWE); cases 5 and 6, aneurysms with circumferential wall enhancement (CAWE)

Blinded to patient information, two experienced neuroradiologists (both with more than 10 years of experience in neurovascular imaging) independently compared the post-contrast images of each aneurysm with the precontrast ones to categorize the AWE patterns into 3 groups: (a) no AWE (NAWE, no enhancement of the wall compared with precontrast scan); (b) partial AWE (PAWE, only part of the wall presented as enhanced); and (c) circumferential AWE (CAWE, the whole wall presented as enhanced). Discordances were resolved by a third reader (20 years of experience in neurovascular imaging). Representative cases of NAWE, PAWE, and CAWE are shown in Fig. 1.

### Hemodynamic Analysis

3DRA was performed by the Artis zee biplane angiographic system (Siemens, VC14, Germany). A 5sDSA acquisition protocol was adopted, and a total of 18 ml of contrast agent was injected through the internal carotid artery (ICA) or vertebral artery in a rate of 3 ml/s. During 5-s acquisition after a delay of 1 s, 200 rotation of C-arm was performed to obtain 133 frames. All the acquired 5sDSA data were transferred to a *syngo* X Workplace (Siemens, VB15, Germany) for reconstruction of the 3D vessel tree and exported as stereolithography (STL) format.

The 3D models were segmented and smoothed by the Meshmixer 3.2 software (Autodesk Inc., San Francisco, CA, USA) and then imported into ICEM CFD 11.0 (ANSYS, Lebanon, NH) to create volume grids for fluid dynamics simulation. The number of total elements of each model was approximately between 800,000 and 1,200,000. A pulsatile velocity waveform was obtained by transcranial Doppler (TCD) from a normal subject [12].

CFD simulations were performed by CFX 11.0 (ANSYS). The vessel was considered a rigid wall with no-slip boundary conditions. The governing equations underlying the calculation were the Navier-Stokes formulations, with an assumption of a laminar and incompressible blood flow. The density and dynamic viscosity of it were specified as *ρ* = 1050 kg/m^3^ and *μ* = 0.00345 Pa s. The inlet was imposed by the pulsatile velocity profile obtained by TCD, and the outlet was defined as opening boundary condition with zero static pressure. We discretized the entire cardiac cycle of 0.8 s by a time step of 0.001 s for numeric simulation. Three continuous cardiac cycles were simulated to ensure the numeric stability of the simulation, and the last cycle was taken as output.

Several hemodynamic parameters were included in this study: wall shear stress (WSS), percentage of low WSS area (LSA), oscillatory shear index (OSI), pressure and relative residence time (RRT). To allow comparison among different patients, normalized WSS and pressure were calculated by dividing the time-averaged WSS or pressure of the aneurysm dome to the counterpart of its parent artery. LSA, defined as the areas of the aneurysm wall exposed to a WSS below 10% of the mean WSS of parent artery, was then normalized by the dome area. OSI, a non-dimensional parameter, measures the directional change of WSS during the cardiac cycle. RRT, a combination of WSS and OSI, reflects the residence time of blood near the wall:

1 $$ \mathrm{WSS}=\frac{1}{T}{\int}_0^T\left|{\mathrm{wss}}_{\mathrm{i}}\right| dt $$

2 $$ \mathrm{OSI}=\frac{1}{2}\left\{1-\frac{\left|\underset{0}{\overset{T}{\int }}{\mathrm{wss}}_{\mathrm{i}} dt\right|}{\underset{0}{\overset{T}{\int }}\left|{\mathrm{wss}}_{\mathrm{i}}\right| dt}\right\} $$

3 $$ \mathrm{RRT}=\frac{1}{\left(1-2\times OSI\right)\times WSS}=\frac{1}{\frac{1}{T}\left|{\int}_0^T{wss}_i dt\right|} $$

where wss_i_ is the instantaneous WSS vector and *T* is the duration of the cycle. The OSI was averaged over the dome area.

### Morphological Analysis

Morphological parameters were defined as previously reported [13]. The size of the aneurysm dome was defined as the maximum diameter of the aneurysm dome. Dome height was the longest dimension from the neck to the dome tip, and dome width was measured perpendicular to the dome height. Aspect ratio was computed by dividing dome height by neck width. Size ratio was calculated by dividing size by the average diameter of parent arteries and dome-to-neck ratio by dividing size by neck width. Bottleneck factor was defined as the ratio of dome width to neck width. Height-to-width parameter was determined using the ratio between the height and width of the aneurysm. Inflow angle was the angle between inflow and the aneurysm’s main axis from the center of the neck to the tip of the dome. Irregular shape was defined as small bleb(s) or secondary aneurysm(s) were protruding from the aneurysm fundus or the aneurysm fundus was clearly bi- or multilobular.

### Statistics

Statistical analysis was performed with Microsoft Excel 2010 and SPSS 20.0 (IBM Corp, Armonk, NY). Variables were expressed as median (interquartile range) or number of patients (%) as appropriate. *p* < 0.05 (two-sided) was the criterion for statistical significance.

To investigate the differences of baseline, morphological, and hemodynamic data between AWE patterns, the chi-square test or Kruskal-Wallis *H* test was performed for cross-tabulation or measured data, respectively. Then, the significant variables in univariate analysis were further analyzed using an ordinal logistic regression to identify independent risk factors for AWE patterns.

Finally, the rupture risk of each aneurysm was calculated by the PHASES score, which is based on population (*P*, scores 0–5), hypertension (*H*, scores 0–1), age (*A*, scores 0–1), size (*S*, scores 0–10), early history of subarachnoid hemorrhage (*E*, scores 0–1), and location (*S*, scores 0–4) [14]. As no definition of the PHASES score for the Chinese population exists, we calculated the scores by assuming the rupture risk of Chinese population was equal to North American and European population (score 0). For example, a 75-year-old patient with a 15-mm middle cerebral artery (MCA) aneurysm and without hypertension or subarachnoid hemorrhage history will be scored as population (0) + hypertension (0) + age (1) + size (6) + early subarachnoid hemorrhage (0) + site (2) = 9. The distributions of morphological, hemodynamic, and AWE characteristics in different PHASES score sections (0–4, 5–7, and 8–) were investigated to reveal their correlations with aneurysm rupture risk by the chi-square test or Kruskal-Wallis *H* test.

## Results

### General Characteristics of the Patients and Aneurysms

Baseline characteristics of the patients and UIAs are shown in Table 1. The age of the 57 patients (22 males and 35 females) ranged from 36 to 80 years, with a mean age of 58.4 years. Twenty-six patients (45.6%) had hypertension, 6 (10.5%) had diabetes mellitus, 7 (12.3%) were current smokers, and 6 (10.5%) had early history of subarachnoid hemorrhage. Multiple aneurysms were presented in 13 (22.8%) patients.

**Table 1:** Baseline, morphological, and hemodynamic characteristics in aneurysms with different wall enhancement patterns

Variable	Non-AWE, *N* = 39	Partial AWE, *N* = 14	Circumferential AWE, *N* = 12	Statistical methods	*p* value
Age (years)	56 (52.63)	59 (56.70)	60 (55.69)	Kruskal-Wallis *H*	0.218
Male	17 (43.6)	5 (35.7)	3 (25.0)	Chi-square	0.497
Hypertension	19 (48.7)	6 (42.9)	8 (66.7)	Chi-square	0.443
Earlier SAH	3 (7.7)	1 (7.1)	2 (16.7)	Chi-square	0.614
Diabetes	4 (10.3)	1 (7.1)	2 (16.7)	Chi-square	0.727
Smoking	5 (12.8)	2 (14.3)	2 (16.7)	Chi-square	0.943
Familiar SAH	4 (10.3)	4 (28.6)	1 (8.3)	Chi-square	0.195
Location				Chi-square	0.309
ICA	22 (56.4)	4 (28.6)	4 (33.3)		
Acom/ACA	3 (7.7)	3 (21.4)	1 (8.3)		
MCA	9 (23.1)	5 (35.7)	3 (25.0)		
Pcom/PC	5 (12.8)	2 (14.3)	4 (33.3)		
Multiplicity	13 (33.3)	4 (28.6)	4 (33.3)	Chi-square	0.945
Size (mm)	5.31 (4.55, 7.63)	7.83 (5.81, 9.42)	8.83 (7.59, 10.94)	Kruskal-Wallis *H*	< 0.001
Aspect ratio	0.90 (0.83, 1.10)	1.07 (0.82, 1.35)	1.18 (1.02, 2.42)	Kruskal-Wallis *H*	0.011
Size ratio	2.26 (1.64, 2.79)	3.23 (2.03, 5.02)	3.75 (3.01, 5.29)	Kruskal-Wallis *H*	< 0.001
Height-width ratio	0.81 (0.71, 0.93)	0.88 (0.75, 0.95)	0.92 (0.87, 1.03)	Kruskal-Wallis *H*	0.064
Bottleneck index	1.04 (0.95, 1.30)	1.16 (1.09, 1.33)	1.25 (1.14, 2.00)	Kruskal-Wallis *H*	0.013
Dome-to-neck ratio	1.18 (1.03, 1.45)	1.37 (1.20, 1.59)	1.63 (1.28, 2.53)	Kruskal-Wallis *H*	0.007
Inflow angle (°)	126 (105, 138)	129 (104, 156)	130 (112, 164)	Kruskal-Wallis *H*	0.337
Irregular shape	9 (23.1)	8 (57.1)	8 (66.7)	Chi-square	0.007
Normalized WSS	0.70 (0.61, 0.96)	0.62 (0.46, 0.81)	0.43 (0.28, 0.50)	Kruskal-Wallis *H*	0.001
Min_WSS (Pa)	4.98 (2.73, 6.90)	2.90 (1.89, 4.35)	2.75 (1.65, 4.09)	Kruskal-Wallis *H*	0.014
Max_WSS (Pa)	8.12 (5.30, 10.29)	5.14 (4.51, 6.75)	8.78 (6.93, 10.73)	Kruskal-Wallis *H*	0.101
Low WSS area	0.00 (0.00, 0.02)	0.00 (0.00, 0.02)	0.05 (0.01, 0.22)	Kruskal-Wallis *H*	0.003
Oscillatory shear index	0.02 (0.01, 0.05)	0.02 (0.01, 0.04)	0.04 (0.02, 0.07)	Kruskal-Wallis *H*	0.318
Pressure	1.05 (1.02, 1.10)	1.06 (1.01, 1.13)	1.02 (1.00, 1.12)	Kruskal-Wallis *H*	0.478
Relative residence time	0.18 (0.12, 0.39)	0.28 (0.15, 0.48)	0.33 (0.29, 0.56)	Kruskal-Wallis *H*	0.022

*SAH*, subarachnoid hemorrhage; *ICA*, internal carotid artery; *Acom*, anterior communicating artery; *ACA*, anterior cerebral artery; *MCA*, middle cerebral artery; *Pcom*, posterior communicating artery; *PC*, posterior circulation; *WSS*, wall shear stress

The aneurysmal size ranged from 2.6 to 19.6 mm, with 25 (38.4%) presented with irregular shape. Thirty (46.2%) aneurysms located in ICA, 17 (26.2%) aneurysms in MCA, 6 (9.2%) aneurysms in posterior circulation, 5 (7.7%) in posterior communicating artery, 6 (9.2%) in anterior communicating artery, and 1 (0.2%) in anterior cerebral artery.

### Morphological and Hemodynamic Characteristics Related to AWE

Of the total 65 UIAs, NAWE was detected in 39 (60.0%) lesions, PAWE was detected in 14 (21.5%) lesions, and CAWE in 12 (18.5%) lesions. Discordances of interpreting AWE patterns occurred in 7 lesions, of which in the final interpretation, 5 were determined as PAWE and 2 as CAWE. In the univariate analysis, no statistical difference of baseline parameters (age, gender, and medical history) was observed between aneurysms with different AWE patterns. Of the morphological and hemodynamic variables, aneurysm size (*p* < 0.001), aspect ratio (*p* = 0.011), size ratio (*p* < 0.001), bottleneck factor (*p* = 0.013), dome-to-neck ratio (*p* = 0.007), irregular shape (*p* = 0.007), normalized WSS (*p* = 0.001), minimum WSS (*p* = 0.014), LSA (*p* = 0.003), and RRT (*p* = 0.022) showed significant differences among the 3 groups (Fig. 1).

Multivariate analysis by ordinal logistic regression was performed to identify the independent factors that affected the AWE patterns. Finally, size (odds ratio = 7.46; *p* = 0.012), size ratio (odds ratio = 12.90; *p* = 0.004), and normalized WSS (odds ratio = 0.11; *p* = 0.018) were revealed to be independently associated with the AWE pattern of aneurysms (Table 2).

**Table 2:** Multivariate analysis of morphological and hemodynamic variables that associated with aneurysmal wall enhancement

Variable	Odds ratio	95% CI	*p* value
Size	7.46	1.56–35.77	0.012
Aspect ratio	0.26	0.05–1.50	0.133
Size ratio	12.90	2.28–72.97	0.004
Bottleneck factor	1.66	0.31–8.96	0.538
Dome-to-neck ratio	1.18	0.17–8.17	0.868
Irregular shape	1.32	0.35–4.98	0.687
Normalized WSS	0.11	0.02–0.69	0.018
Low WSS area	0.93	0.19–4.59	0.982
Minimum WSS	1.25	0.23–6.88	0.796
Relative residence time	0.21	0.04–1.18	0.076

*WSS*, wall shear stress

### Morphological, Hemodynamic, and AWE Patterns of High Rupture Risk

**Fig. 2:**
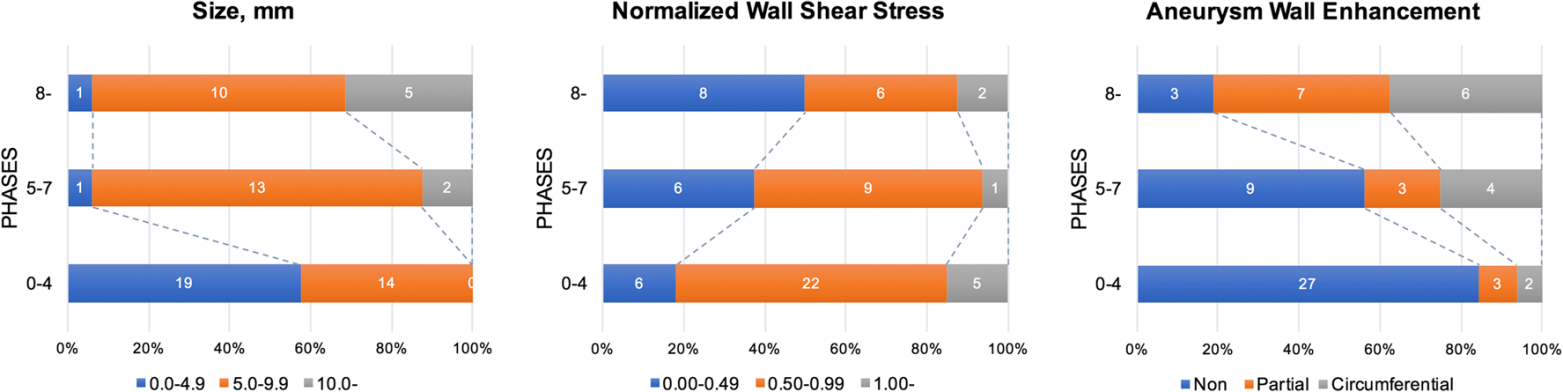
Distributions of aneurysmal size, normalized wall shear stress, and wall enhancement in different PHASES score sections

The PHASES score was calculated for each case to assess the rupture risk. According to the PHASES study, score 5 and score 8 are corresponding to the 5-year rupture probability of 1% and 3%, respectively [14]. We therefore divided the global score into 3 sections: 0–4, 5–7, and ≥ 8. Of the 65 aneurysms, 33 scored 0–4, 16 scored 5–7, and 16 scored 8. With increasing score, the morphology of aneurysms showed a larger size pattern, with significantly larger size, size ratio, bottleneck factor, and dome-to-neck ratio. On the other hand, the distribution of hemodynamic variables revealed a lower and oscillatory trend, with statistically lower normalized WSS, higher LSA and OSI, and prolonged RRT. The distribution of AWE patterns was significantly varying in different score sections (*p* < 0.001). Of the 33 aneurysms that scored 0–4, 29 (87.9%) presented with NAWE, and no CAWE was observed in this section. The proportion of NAWE dropped to 56.3% (9/16) in the section of score 5–7 and to 18.8% (3/16) in the section of score ≥ 8, while the frequencies of PAWE and CAWE increased as the score increased (Table 3 and Fig. 2).

**Table 3:** Morphology, hemodynamics, and aneurysmal wall enhancement in aneurysms with different PHASES scores

Variable	Scores 0–4, *N* = 33	Scores 5–7, *N* = 16	Score 8–, *N* = 16	Statistical methods	*p* value
Morphological pattern					
Size (mm)	4.74 (4.06, 6.74)	7.73 (6.42, 9.00)	9.20 (7.80, 10.94)	Kruskal-Wallis *H*	< 0.001
Aspect ratio	0.96 (0.84, 1.11)	0.94 (0.83, 1.30)	1.17 (0.88, 1.66)	Kruskal-Wallis *H*	0.154
Size ratio	2.00 (1.19, 2.42)	2.71 (2.43, 3.32)	4.64 (3.45, 5.80)	Kruskal-Wallis *H*	< 0.001
Bottleneck factor	1.03 (0.94, 1.25)	1.20 (1.08, 1.61)	1.18 (1.10, 1.80)	Kruskal-Wallis *H*	0.010
Dome-to-neck ratio	1.20 (1.03, 1.40)	1.26 (1.07, 1.71)	1.53 (1.29, 2.17)	Kruskal-Wallis *H*	0.005
Height-to-width ratio	0.86 (0.76, 1.02)	0.81 (0.70, 0.91)	0.90 (0.79, 1.03)	Kruskal-Wallis *H*	0.280
Inflow angle (°)	122 (101, 134)	127 (106, 137)	142 (117, 164)	Kruskal-Wallis *H*	0.020
Irregular shape	7 (21.2)	7 (43.8)	11 (68.8)	Chi-square	0.005
Hemodynamic pattern					
Normalized WSS	0.72 (0.59, 0.97)	0.62 (0.46, 0.73)	0.48 (0.28, 0.67)	Kruskal-Wallis *H*	0.014
Low WSS area	0.00 (0.00, 0.01)	0.01 (0.00, 0.03)	0.03 (0.00, 0.22)	Kruskal-Wallis *H*	0.025
Oscillatory shear index	0.02 (0.01, 0.03)	0.04 (0.02, 0.06)	0.04(0.02, 0.06)	Kruskal-Wallis *H*	0.006
Pressure	1.03 (1.01, 1.09)	1.06 (1.02, 1.19)	1.07 (1.02, 1.15)	Kruskal-Wallis *H*	0.210
Relative residence time	0.17 (0.11, 0.32)	0.30 (0.16, 0.46)	0.44 (0.29, 0.60)	Kruskal-Wallis *H*	0.001
Wall enhancement				Chi-square	< 0.001
Non-AWE	29 (87.9)	9 (56.3)	3 (18.8)		
Partial AWE	4 (12.1)	3 (18.8)	7 (43.8)		
Circumferential AWE	0 (0.0)	4 (25.0)	6 (37.5)		

*WSS*, wall shear stress; *AWE*, aneurysm wall enhancement

## Discussion

Previous studies have shown the presence of inflammatory cell infiltrations and inflammatory cytokines in the aneurysm wall and finally their association with critical weakening of aneurysm wall and rupture [7]. Hemodynamics is believed to be the initiating factor for this inflammatory reaction [8]. Although various attempts have been made, from flow-induced animal models to histological analysis of human tissues, the link between hemodynamics and wall inflammation is yet not fully understood [15]. It is also unclear how to translate our knowledge on this underlying mechanism into improvement of clinical aneurysm rupture stratification.

We hypothesize that the aneurysm wall will exhibit stronger degeneration, possibly greater inflammation, the longer it is exposed to adverse hemodynamic conditions. Larger aneurysms, which will have grown while being exposed to these adverse hemodynamic conditions, will therefore exhibit a wall that displays higher degeneration or inflammation. If grown under adverse hemodynamic conditions, aneurysms may show an irregular shape so that we expect a correlation of irregularity with rupture risk and inflammation.

The ability to visualize and quantify inflammation in vivo will aid in testing above hypothesis and consequently in developing a tool for clinical decision-making applicable to UIAs. Hasan et al. [16] have investigated the feasibility for imaging wall inflammation using ferumoxytol-enhanced VW-MRI. As ferumoxytol might be not available for routine MRI scans, AWE using gadolinium-based contrast agents was suggested to be an alternative to detect aneurysm wall inflammatory reactions [10, 11, 17]. Several studies have revealed the value of AWE in discriminating rupture status of aneurysms, in which aneurysm size and irregular shape were correlated with the presence of AWE and rupture risk, thereby supporting the hypothesis we have formulated above [11, 17]. According to a recent meta-analysis, VWI presented with a relatively high sensitivity of 95.0% and a moderate specificity of 62.7% in identifying unstable aneurysms [18]. To further improve the specificity of VWI in clinical use, combining analysis with other rupture risk–related factors, like aneurysm hemodynamics, might be an alternative strategy.

The role of aberrant hemodynamics in aneurysm formation, growth, and rupture has been widely investigated, especially after the application of CFD technique in this field. WSS has been found to be the key hemodynamic factor in aneurysm progression. Our previous studies demonstrated lower WSS to be more common in aneurysms with high rupture risk [19, 20]. At the same time, significantly lower WSS was observed in UIAs with AWE in this study. These results are consisted with previous studies, in which endothelial cell loss and differential expression of inflammatory cytokines were revealed in areas of low WSS [21]. Leukocyte transmigration into the wall will be enhanced by an increased blood residence time that causes aneurysm enlargement, which corroborates with the observed increased RRT in aneurysms with AWE [22].

The relationship of the hemodynamic and morphological parameters with the PHASES score, i.e., that aneurysms tend to present with lower WSS and more intense AWE, suggests a correlation of hemodynamics and inflammation with high rupture risk in line with our hypothesis. A recent population-based study suggested that aneurysm with a low PHASES score was still associated with a non-negligible likelihood of rupture [23]. This is also reflected in our data where some aneurysms of small size still presented with aberrant WSS and AWE. In the context of our findings, these aneurysms, regardless of size, may possess a degenerated wall with higher inflammation and therefore show a higher rupture risk.

In summary, our retrospective study lends strong support to an aneurysm growth and rupture model, in which aberrant hemodynamic conditions cause or correlate with irregular growth, resulting in an inflamed and degenerated or weakened wall prone to rupture. These findings also highlight the potential role of VWI in aneurysm rupture prediction and suggest that wall enhancement should be considered when building aneurysm rupture models. The combination of morphology, hemodynamics, and aneurysmal wall characteristics might further improve the accuracy of clinical aneurysm rupture prediction. A prospective study including a larger number of cases is warranted to confirm these findings and to define the models based on multiple dimensional data that can be used in a clinical tool for treatment decision-making.

The present study has well-known limitations. Firstly, the retrospective design and the relatively small sample size from a single center might generate bias for the data collection and statistics analysis. Secondly, although 3DRA images were used to obtain the most accurate patient-specific models for CFD analysis, several approximations were made as in previous CFD studies. The blood flow was assumed Newtonian, the vascular walls were assumed rigid, and physiologic pulsatile flow conditions from healthy subjects were used, all of which may introduce small errors in the calculation of hemodynamic parameters. Thirdly, although the PHASES score has been applied in several studies, it is not a gold standard to discriminate IA with high rupture risk and requires more prospective evidences. In addition, no Chinese population was included in the original study defining the PHASES score, so that in this study, the rupture risk of the Chinese population was assumed equal to other populations, which might cause some bias. Also, image artifacts may exist in the MRI AWE images caused by slow moving blood mimicking AWE.

## Conclusion

Morphological and hemodynamic characteristics were closely associated with the presence of AWE. UIAs with a higher rupture risk presented with significant larger size, lower wall shear stress, and more intense AWE, which might support the interaction between morphology, hemodynamics, and inflammation in aneurysm rupture prediction.
